# Supplementary material for: Identifying grain boundary and intragranular pinning centres in Sm2(Co,Fe,Cu,Zr)17 permanent magnets to guide performance optimisation
Source: Nat Commun. 2025 Dec 20;16:11335. doi: 10.1038/s41467-025-67773-7 (PMC12722751; doi:10.1038/s41467-025-67773-7)
Supplement: Supplementary file 1 — Supplementary Information [file 41467_2025_67773_MOESM1_ESM.pdf]

Supplementary Material

Identifying grain boundary and intragranular pinning centres in $Sm_2(Co,Fe,Cu,Zr)_{17}$ permanent magnets to guide performance optimisation

Stefan Giron[1], Nikita Polin[2], Esmaeil Adabifiroozjaei[3], Yangyiwei Yang[1], Fernando Maccari[1], András Kovács[4], Trevor P. Almeida[5], Dominik Ohmer[1,6], Kaan Üstüner[6], Alaukik Saxena[2], Matthias Katter[6], Iliya A. Radulov[1], Christoph Freysoldt[2], Rafal E. Dunin-Borkowski[4], Michael Farle[7], Karsten Durst[1], Hongbin Zhang[1], Lambert Alff[1], Katharina Ollefs[7], Bai-Xiang Xu[1], Oliver Gutfleisch[1,2], Leopoldo Molina-Luna[3,#], Baptiste Gault[1,8,9#], Konstantin P. Skokov[1,#]

[1] Institute of Materials Science, Technische Universität Darmstadt, 64287 Darmstadt, Germany

[2] Max Planck Institute for Sustainable Materials, Düsseldorf 40237, Germany

[3] Advanced Electron Microscopy Division, Institute of Material Science, Technical University of Darmstadt, 64287 Darmstadt, Germany

[4] Ernst Ruska-Centre for Microscopy and Spectroscopy with Electrons, Forschungszentrum Jülich, Jülich 52425, Germany.

[5] SUPA, School of Physics and Astronomy, University of Glasgow, Glasgow G12 8QQ, United Kingdom

[6] VACUUMSCHMELZE GmbH & Co. KG, 63450 Hanau, Germany

[7] Faculty of Physics and Center for Nanointegration (CENIDE), Universität Duisburg-Essen, 47048 Duisburg, Germany

[8] Department of Materials, Royal School of Mines, Imperial College London, London, UK.

[9] present address: Univ Rouen Normandie, CNRS, INSA Rouen Normandie, Groupe de Physique des Matériaux, UMR 6634, F-76000 Rouen, France

[#] correspondonding authors:

Baptiste Gault: baptiste.gault1@univ-rouen.fr

Leopoldo Molina-Luna : leopoldo.molina-luna@aem.tu-darmstadt.de

Konstantin P. Skokov: konstantin.skokov@tu-darmstadt.de

1 Magnetic Measurements and Microscopy

Magnetization curves of the two investigated samples are compared in **Fig. S1**. The initial curves show the expected pinning type behaviour, with sample A having a higher initial susceptibility (the initial magnetic susceptibility was defined as the slope of the magnetization curve). Remanent polarization of sample A is 1.18T, whereas for sample optimally annealed sample B it is 1.97T. For sample A, a small step-like drop at low fields is present and a pronounced demagnetization with increasing fields above 1.0 T. Moreover, the quick demagnetization is starting at lower fields. For the reference sample B both, the delay of the initial curve as well as the continuous demagnetization exceed the behaviour of the sample A.

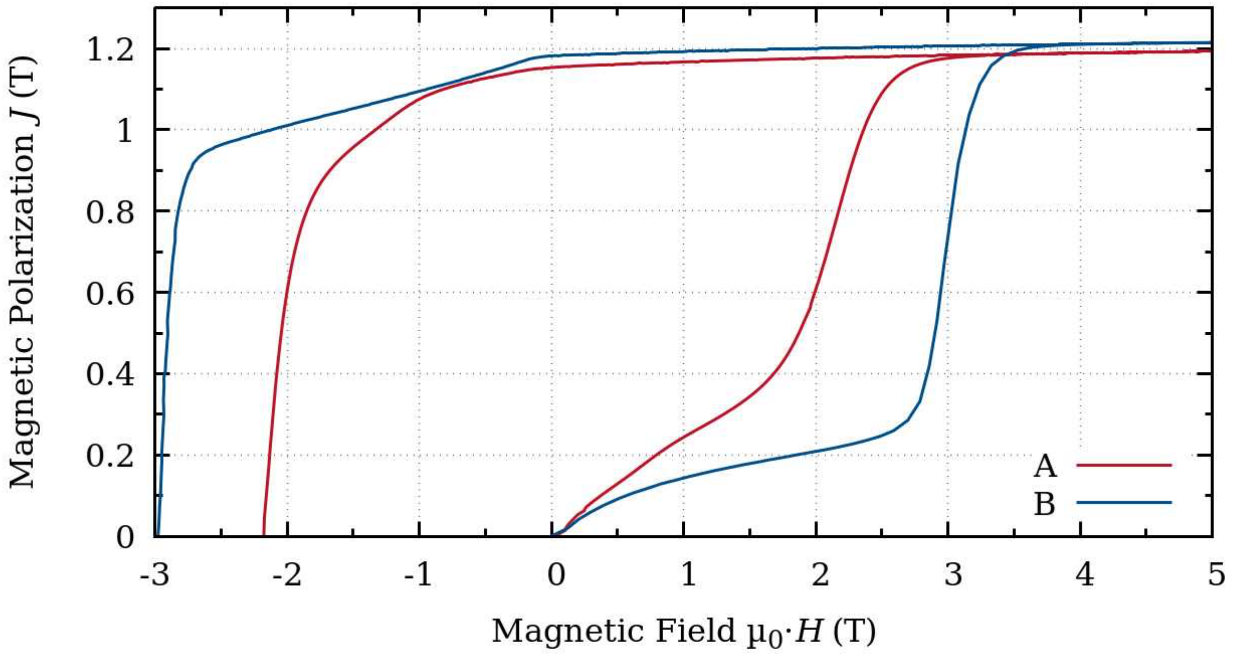

Fig. S1 Initial curve and demagnetization of the samples A and B after applying a field of 14 T. All samples show a pinning-like initial magnetization curve with significantly higher initial susceptibility and weaker pinning in the case of A. At demagnetizing fields as small as 0.3 T a shoulder appears for the sample A, lowering its BH_{max}. Contrary to the reference magnet, sample A shows a two-step demagnetization with increased susceptibilities in demagnetizing fields larger than approximately 1.0 T.

The microstructures and domain patterns of the samples A and B are directly compared in **Figure S2**. Sample A exhibits regions with finer cells and magnetic domains, located close to the GBs within the grains. When saturating and afterwards demagnetizing the sample, these regions again act as weak spots, referred to as low Hc regions, showing magnetization reversal before any other area is affected (cp. **Fig. S2c**). No such low Hc regions are observed in the sample B. Apart from this difference, the microstructures of both samples are closely similar, with compatible grain sizes of 200µm for sample A and 220µm for sample B, see **Table S1**.

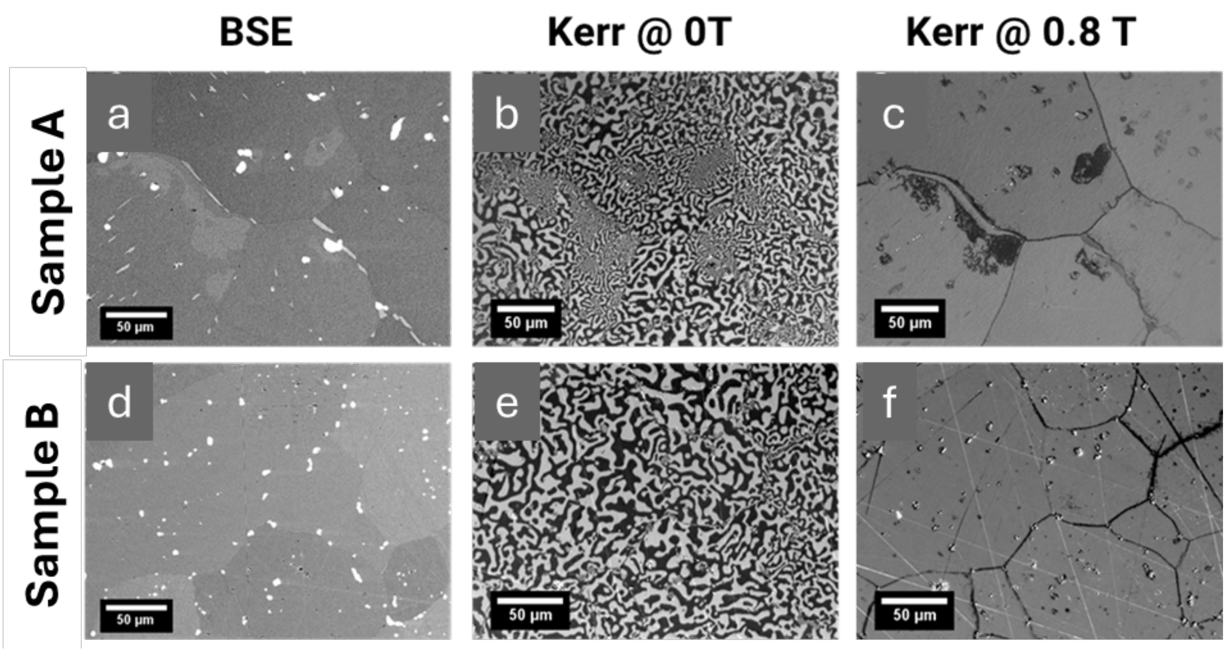

Fig. S2 SEM images using BSE (**(a)**, **(d)**) and Kerr microscopy images of the thermally demagnetized state (**(b)**, **(e)**) and after applying an external field of 0.8 T (**(c)**, **(f)**) to the saturated samples A (top row), B (bottom row), respectively. The same sample regions are shown for SEM and Kerr microscopy.

Table S1 Average grain size d_g and average cell size d_c for A and B samples in the regular and distorted areas respectively.

sample	grain size d_g / µm	area	cell size d_c / nm
A	200	high H_c	272(62)
		low H_c	238(31)
B	220	high H_c	275(45)

A more detailed view via High Resolution (HR) SEM (**Fig. S3**) reveals smaller cell sizes in the low coercivity regions of sample A. The interface of a low and a high coercivity region show continuous magnetic domains and the difference in domain size in MFM (**Fig. S5**).

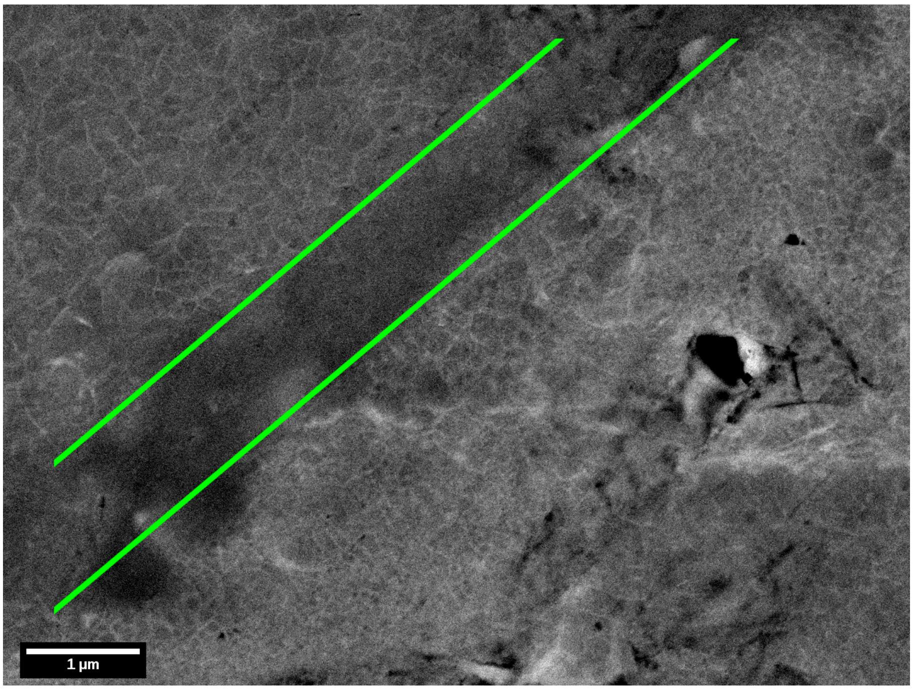

Fig. S3 HR-SEM image of A sample of a high H_c region on the left side of the grain boundary (marked by green lines) and a low H_c region on the right. A finer cell structure is visible in the low H_c region. The surface is perpendicular to the *c*-axis of the 2:17 phase.

Table S2 Average concentrations (wt.%) of the elements in the high and low coercivity regions in in sample A, determined by SEM-EDX and also derived from APT scans are given in the two bottom rows for comparison.

sample	area	Sm	Fe	Co	Cu	Zr
A (EDX)	high H_c	11.9(13)	24.3(10)	57.6(10)	4.7(29)	1.5(38)
	low H_c	12.6(13)	23.0(10)	57.4(10)	5.4(26)	1.6(40)
	precipitate	13.3(12)	15.3(11)	58.2(10)	6.3(25)	6.9(23)
A (APT)	high H_c	11.3(1)	24.7(2)	58.1(1)	4.5(1)	1.3(1)
	low H_c	12.3(2)	22.9(5)	57.4(1)	5.8(4)	1.5(2)

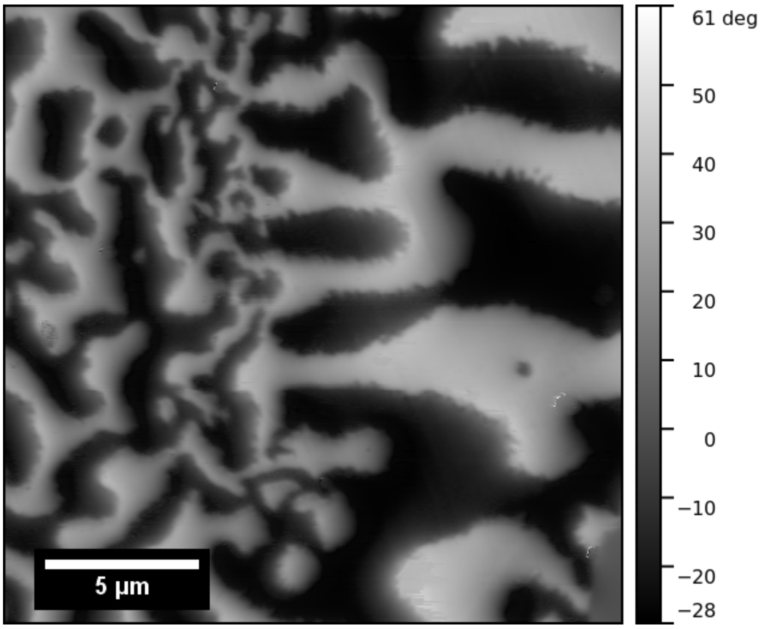

Fig. S4 MFM image of an interface between a low coercivity region (left) and a high coercivity region (right) of sample A.

2 Transmission Electron Microscopy

Phase identification for HAADF-STEM was carried out with the help of theoretical structures as shown with the respective overlays in **Fig. S5**.

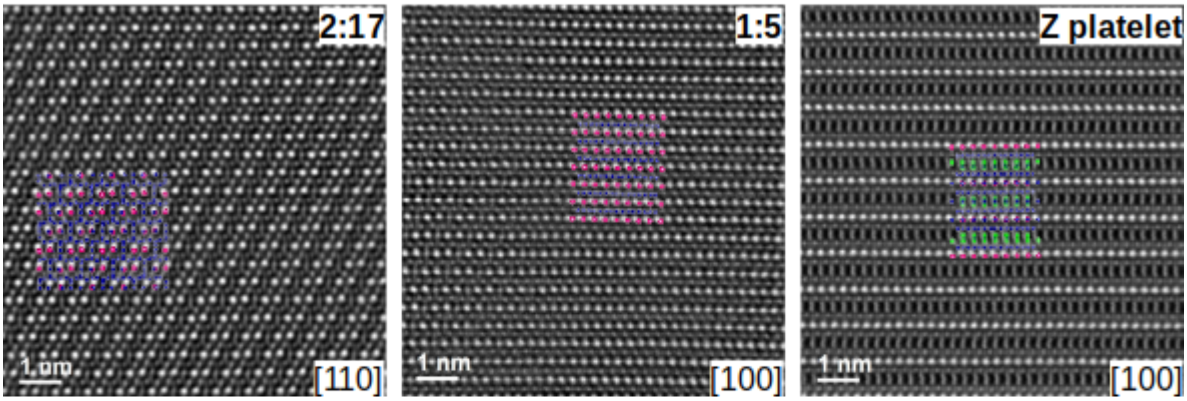

Fig. S5 Atomic stacking of 2:17 R, 1:5, and Z-platelet phases that are used to characterize phases in nanostructures shown in Fig. 8. Images of theoretical structure of phases are superimposed on HAADF images. Pink (Sm), blue (Co), green (Zr).

3 Atom Probe Tomography

Table S3 Composition, phase fraction and thicknesses of the respective phases determined from APT data in the high- and low-Hc regions in sample A. The thickness of the 2:17 phases (in italics) was determined from TEM images instead. For the sample B, no standard deviations are given for the volume fraction and total composition as the APT data stems from a single, representative APT measurement. The calculation of compositions is described in detail in Ref.48.

**Sample / Region**	**Phase**	**Volume Fraction** *v* **/ %**	**Thickness** *d* **/ nm**	**Composition** *x* **/ at%**				
				**Sm**	**Co**	**Fe**	**Cu**	**Zr**
A/high H_c	2:17	78.0±1.6	272±62	11.0±0.1	59.3±0.2	27.2±0.1	2.1±0.2	0.4±0.1
	1:5	6.7±0.5	11.7±1.5	14.3±0.3	48.4±1.0	16.5±1.1	20.3±1.8	0.4±0.1
	Z	15.2±1.1	11.7±4.6	10.2±0.8	62.0±1.9	15.4±1.5	3.9±0.7	8.5±1.3
	total	–		11.3±0.1	58.1±0.2	24.7±0.2	4.5±0.1	1.3±0.1
A/low H_c	2:17	65.1±2.8	238±31	11.0±0.1	59.2±0.1	27.7±0.1	1.6±0.1	0.4±0.1
	1:5	14.8±0.9	11.0±2.7	15.0±0.4	52.3±0.6	17.0±0.9	15.3±1.1	0.4±0.1
	Z	20.1±1.9	10.8±2.8	11.7±0.5	60.7±0.7	16.4±1.1	3.8±0.4	7.4±1.2
	total	–		12.3±0.2	57.4±0.2	22.9±0.5	5.8±0.4	1.5±0.2
B/grain interior	2:17	76.6	-	11.2±0.1	59.3±0.2	26.6±0.2	2.4±0.6	0.5±0.1
	1:5	7.53	-	13.4±0.7	49.9±2.3	18.4±1.2	17.6±3.5	0.7±0.2
	Z	15.82	-	10.4±0.7	59.5±1.3	16.5± 1.1	5.0±0.5	8.5±0.9
	total	-		11.3	58.3	24.4	4.7	1.3

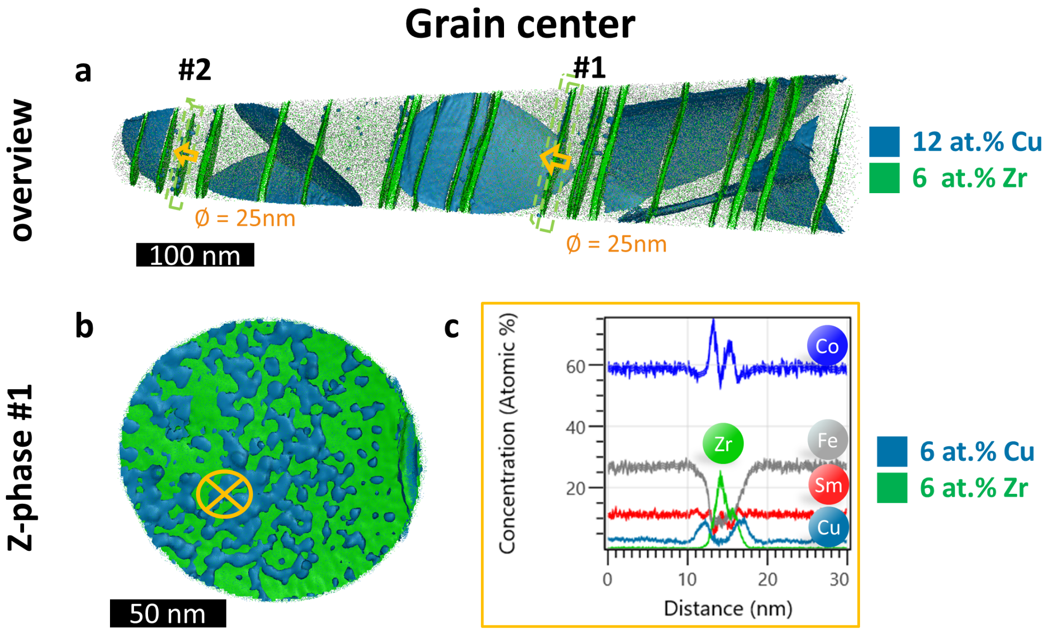

Fig. S6 APT data for the grain center of the reference sample B. **a** 3D reconstruction showing the geometrical distribution of the 1:5 phase (blue, isoconcentration value 12 at.% Cu) and the Z-phase (green, isoconcentration value 6 at.% Zr). Green dashed lined boxes indicate a representative a rotated 3D reconstruction of the Z-platelets (**b**). 1D composition profile is measured perpendicular to the representative Z-platelet (**c**) as indicated by the orange arrows and crossed circles in **a**,**b**. The thinner Z-platelets with thickness around 5 nm possess Cu-rich coating layers at their interfaces, but no Cu-enriched centers as found in the thicker Z-platelets.

4 Magnetic imaging

The infocus image in **Fig. S7a** shows an approximately 1 μm wide region between the high-H_c and low-H_c grain, which possess a different microstructure than that in the core grains. The defocus Fresnel image of the same region (**Fig. 4b**) shows characteristic zig-zag-shaped magnetic domain walls in high- and low-H_c regions and a more linear domain wall in the grain boundary region. Based on the domain wall location, the in-plane magnetic field directions are indicated using arrows in **Fig. S7b**. The domain wall imaging in these regions do not show notable differences that can be attributed to the observed high and low coercivity. **Fig. S7c** shows a Fresnel image recorded from a control TEM specimen that was prepared to confirm the domain wall observations in the grain boundary region and extract information on its magnetic properties. The convergent (white) domain wall contrast runs mostly as straight line with small kinks that suggest presence of pinning sites in this region. Note that the grain boundary region lacks the characteristic lamellar structure that is clearly visible in the high and low-Hc regions. The marked rectangular area in **Fig. S7c** was further investigated using off-axis electron holography. **Fig. S7d** shows the in-plane magnetic induction map generated from the total electron optical phase shift. The contour lines and colours indicate the in-plane magnetic field strength and direction. It can be observed that the domain wall runs straight in the grain boundary region and contains kinks once it enters to the high-H_c regions, possible pinned at the 1:5 phase boundaries. The 180° domain wall segment in the grain boundary region allows to measure the domain wall width, as shown in **Fig. S8e**, using the differential of the recorded phase shift using a fitting function of the form $y=y_0-a\tanh((x-x_0)/w)$, where y_0, a, x_0 and w are constants obtained from the fit. The domain wall width δ is defined as $\delta=\pi w$ and is measured to be as 24 ± 4 nm, which is larger than typical domain widths in $SmCo_5$ (2.6 nm) and Sm_2Co_{17} (6.0 nm) based on the literature.

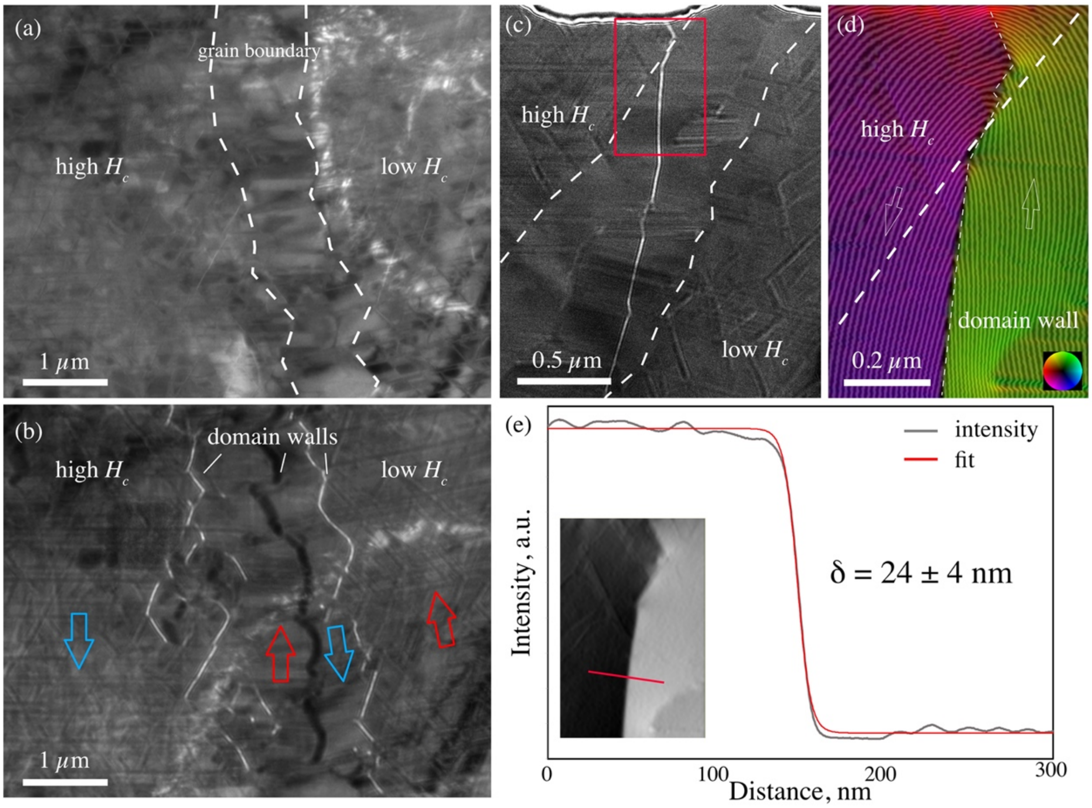

Fig. S7 Magnetic imaging of domain walls with TEM. **(a)** Infocus and **(b)** defocus Fresnel images of the high and low-H_c grains enclosed by the grain boundary region. Dashed lines in (**a**) mark the boundary between the grain boundary region and the high- and low-H_c grains. The black and white contrast lines in (**b**) are the magnetic domain walls. The arrows mark the possible in-plane magnetic field directions between the domains. The defocus value was 0.25 mm. (**c**) Fresnel image of a control specimen showing a magnetic domain wall in the grain boundary region. (**d**) Magnetic induction map of the marked area in (**c**). The contour lines and colour indicate the magnetic field strength and direction at in-plane. The contour spacing is 2π/6 rad. (**e**) Magnetic domain wall width measurement using the (inset) phase shift differential across the 180° domain wall segment. The domain wall width δ is determined to be 24 ± 4 nm.

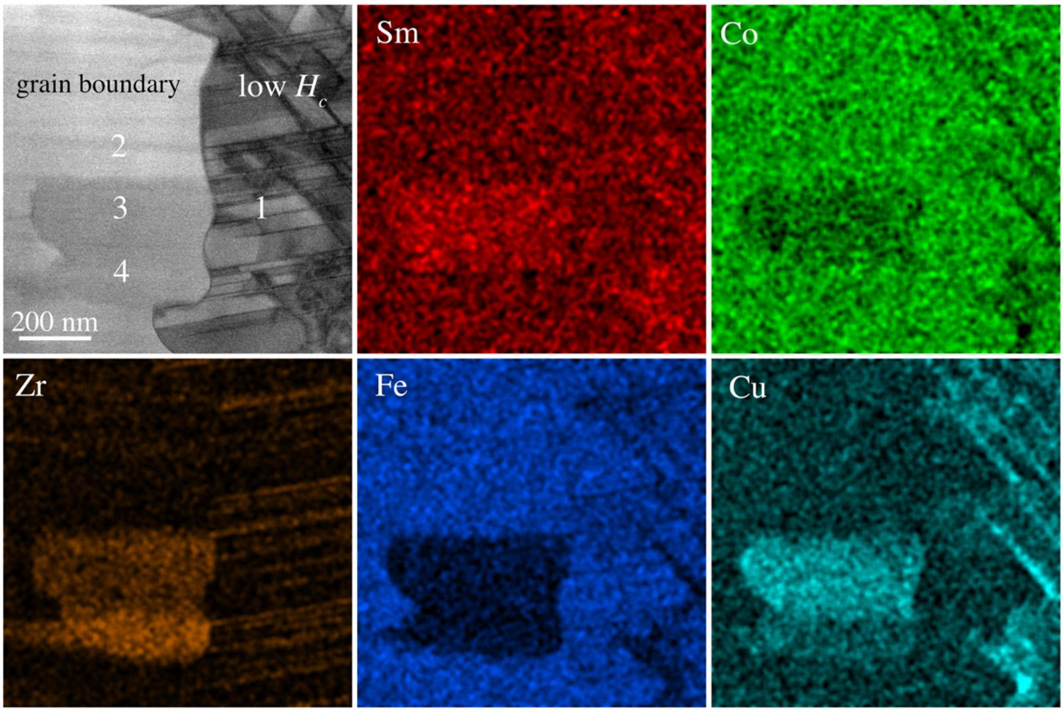

Fig. S8 BF STEM image and the corresponding elemental maps of the grain boundary and low coercivity regions of sample A. Note the lack of the 1:5 Sm-Co cells in the grain boundary region.

Table S4: Chemical compositions in atomic % measured by EDX of regions marked in the STEM BF image in **Fig. S8**.

	Sm	Co	Fe	Cu	Zr
**1**	9	53	21	15	2
**2**	8	54	25	12	1
**3**	11	49	13	21	6
**4**	8	54	15	14	9

5 Micromagnetic Simulation

Micromagnetic simulations were carried out by using the open-source GPU-accelerated finite-difference (FD) program Mumax3[47]. We consider the free energy of the magnetic system as

$$F = F_{ex} + F_{ani} + F_{dm} + F_{ext} \quad \text{(S1)}$$

which is the sum of the exchange energy F_{ex}, the magnetocrystalline anisotropy energy F_{ani}, the magnetostatic (demagnetizing) energy F_{dm}, and the Zeeman energy F_{ext}. Taking the uniaxial anisotropy for all magnetic phases, the free energy of the system with the domain volume Ω and an applied external field $\boldsymbol{H}_{ext}$ can be written as

$$F(M, H_{ext}) = \int_{\Omega} \left[\frac{A}{M_s^2}(\nabla \cdot M)^2 - \frac{K_1}{M_s^2}(u \cdot M)^2 - \mu_0\left(\frac{1}{2}H_{dm} \cdot M + H_{ext} \cdot M\right)\right] dV, \quad \text{(S2)}$$

where $\boldsymbol{u}$ is the uniaxial vector and set to be identical to the magnetocrystalline easy axis c for all coherent phases. $\mathbf{H}_{dm}$ is the demagnetizing field. The exchange parameter A, 1st order uniaxial anisotropy constant K_1, and saturation magnetization M_s are phase-dependent parameters as listed in **Tab. S5**, taken from Ref. [19]. The domain wall energy is also calculated as $\sigma_{dw} = 4\sqrt{AK_1}$. The varying parameters of $Sm(Co_{1-x}Cu_x)_5$ phases versus the copper fraction x are explicitly presented in **Fig. S9a** according to Ref. [49]. The magnetization **M** is then calculated for every increased external field by small increments from

$$\frac{\delta F}{\delta M} = 0, \quad \text{(S4)}$$

where the conjugate gradient (CG) solver is used.

Parameterized nanostructures in a 512x512x4 nm³ domain were generated to recapitulate the spatial characteristics of the underlying phases of the samples in this work, as shown in the **Fig. S9b**. To recapture the domain behaviours in the micromagnetic simulations without artificial effects related to mesh, the FD cell size is chosen as 1 nm, which is smaller than the magnetocrystalline exchange length, *i.e.*, $l_{ex} = \pi\sqrt{A/K_1}$, as also shown in the **Tab. S5**. A grain boundary layer with thickness of 2 nm (as shown in **Fig. S9b**), where magnetocrystalline isotropy is assumed (*i.e.*, $K_1 = 0$), was also introduced to emulate the effects of the grain boundary in reducing the nucleation field to the system [50]. The periodic boundary condition (BC) is applied on the two boundaries perpendicular to the z direction, while the Neumann BC is applied on other boundaries [47].

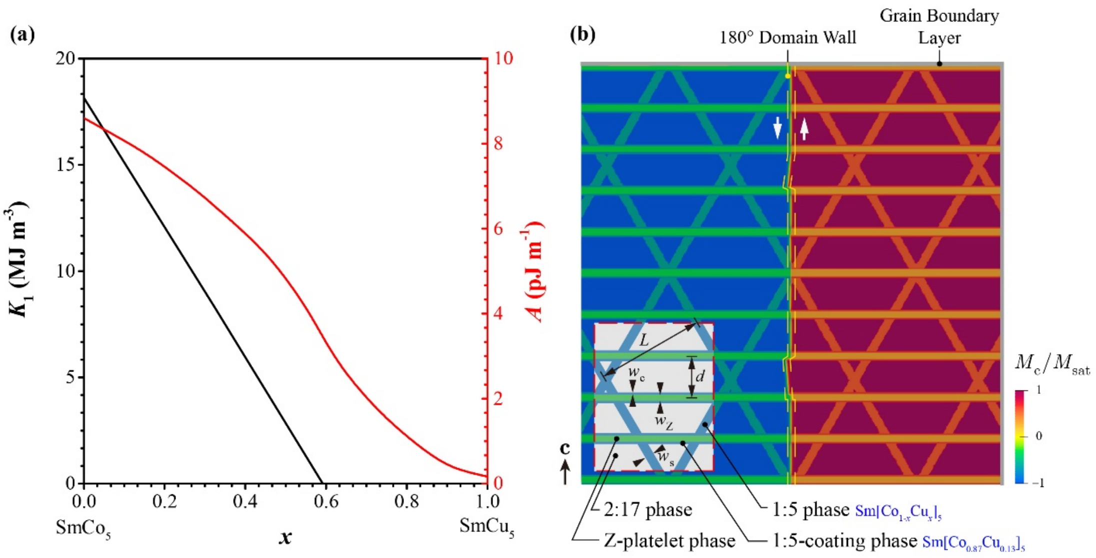

Fig. S9 (a) Dependence of uniaxial anisotropy constant K_1 and exchange constant A from the Cu content x in $Sm(Co_{1-x}Cu_x)_5$ [52]. **(b)** Schematic of the parameterized nanostructure with the 180° domain wall along the easy axis c (taking two-domain IC as an example). An external magnetic field parallel to the c-axis is applied to simulate the reversal behaviour of the nanostructure.

As detailed in **Fig. S10**, four structural parameters are defined: cell size L, distance in between Z-platelets d, thickness of the 1:5 cell wall phase w_s, and thickness of the Z-platelet phase w_Z describing the cellular nanostructure in $Sm_2(Co,Fe,Cu,Zr)_{17}$ magnets following Ref. [19], we introduce the parameter w_c indicating the thickness of the 1:5-coating phase on the Z-platelet phase. Two Cu concentrations $x_{Cu}^{(s)}$ and $x_{Cu}^{(c)}$ are independently considered for the 1:5 cell boundary phase and the 1:5 coating phase, respectively.

Three initial conditions (IC) were studied in the micromagnetic simulation. The two-domain IC with zero overall magnetization was set for the initial magnetizing curve, while nucleated and saturated ICs were chosen for the demagnetization curves. In the nucleated IC, the fully magnetized nanostructure is in contact with a thin boundary layer with reversed magnetization, emulating an existing nucleation of the reversed domain. The domain wall propagation thereby becomes the main process during the demagnetization process, and structural pinning from the nanostructure should have a remarkable effect. On the other hand, demagnetization from the saturated IC ideally forms the other edge case where no nucleus appears in the domain and the whole domain is reversed under a strong applied field. In this sense, the experimentally measured coercive field is expected to lie between the ones from the nucleated and saturated IC, denoted as the lower-bound ($\mu_0 H_{c,L}$) and upper-bound coercive field ($\mu_0 H_{c,U}$), respectively.

To single out the influence of each characteristic, **Fig. S11a-c** sequentially present the simulated initial magnetization and the demagnetization curves on varying w_Z, L and $x_{Cu}^{(s)}$, each for low and high-H_c cases, with and without 1:5-cover layers on top of the Z-platelet phase. The existence of the 1:5-coating generally increases the coercivity (both $\mu_0 H_{c,L}$ and $\mu_0 H_{c,U}$) of the nanostructure with fixed w_Z, L and $x_{Cu}^{(s)}$. Without 1:5-coating phase, varying parameters do not show a distinctive influence on the coercivity. In contrast, the existence of the 1:5-coating phase amplified the influences of modifying w_Z and $x_{Cu}^{(s)}$ on $H_{c,L}$, implying enhanced pinning effects to the domain wall propagation from these two characteristics. However, a beneficial effect on the coercivity by smaller cells may

not be concluded from our set of parameters and only the initial magnetization curve hints on a stronger pinning effect. Remarkably, with 1:5-coating, $\mu_0 H_{c,L}$ increases from 0.8 to 1.6 T when rising $x_{Cu}^{(s)}$ from 18.8 to 28.0 at%. A rather small increase of $\mu_0 H_{c,L}$ from 0.6 to 0.8 T was achieved when rising w_Z from 4 to 16 nm.

To briefly sum up, the existence of 1:5-coating and increased $x_{Cu}^{(s)}$ is the phenomenological reason for the high coercivity of the nanostructure. Increased w_Z also may contribute to enhanced coercivity, but varying L has no evident effect within the scope of this work. **Fig. S11d** presents the combined effects using the proposed two nanostructures, providing good agreement with the experimental observations. By comparing the initial magnetization and the demagnetization curve with nucleated IC of both structures, the high coercivity nanostructure presents a more significant pinning effect on the propagation of domain walls by showing larger increments between consecutive de-pinning steps, resulting in an increased $\mu_0 H_{c,L}$. Meanwhile, it has a relatively higher critical field for the total reversal of the saturated domain, i.e. an increased $\mu_0 H_{c,U}$.

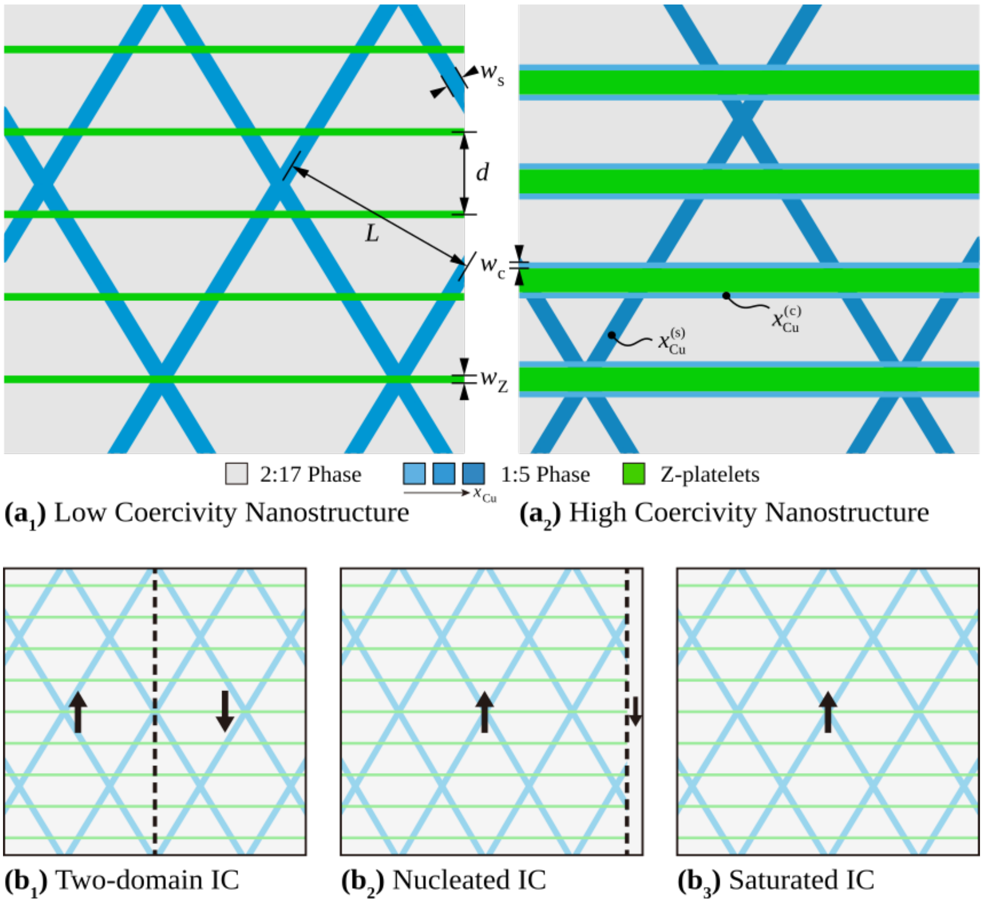

Fig. S10 Proposed nanostructures in the **(a$_1$)** low coercivity and **(a$_2$)** high coercivity regions according to APT and TEM micro- and nanoscale findings. Parameters corresponding to the structural and compositional characteristics of the nanostructure are also denoted. Three different initial conditions (IC) are used for the simulation: the initial magnetization process is modelled with (b1) two-domain IC and the demagnetization process with both **(b$_2$)** nucleated IC and **(b$_3$)** saturated IC.

Table S5 Micromagnetic parameters of the phases used for the simulation [19,52].

**Composition**	**Cu concentration (at%)**	***A* (pJ m^{-1})**	**K_1 (MJ m^{-3})**	**M_s (kA m^{-1})**	**l_{ex} (nm)**	**σ_{dw} (mJ m^{-2})**
$Sm_2(Co,Fe,Cu,Zr)_{17}$	–	19.6	3.9	987.7	7.0	35.0
$Zr_2Sm(Co,Fe,Cu)_9$	–	0.7	1.4	310.4	2.2	4.0
$Sm(Co,Fe,Cu,Zr)_5$	0.0	8.6	18.3	810.8	2.2	50.2
	13.0	7.8	13.7	654.4	2.4	41.3
	18.8	7.3	11.3	580.7	2.5	36.3
	28.0	6.4	7.8	442.3	2.8	28.3

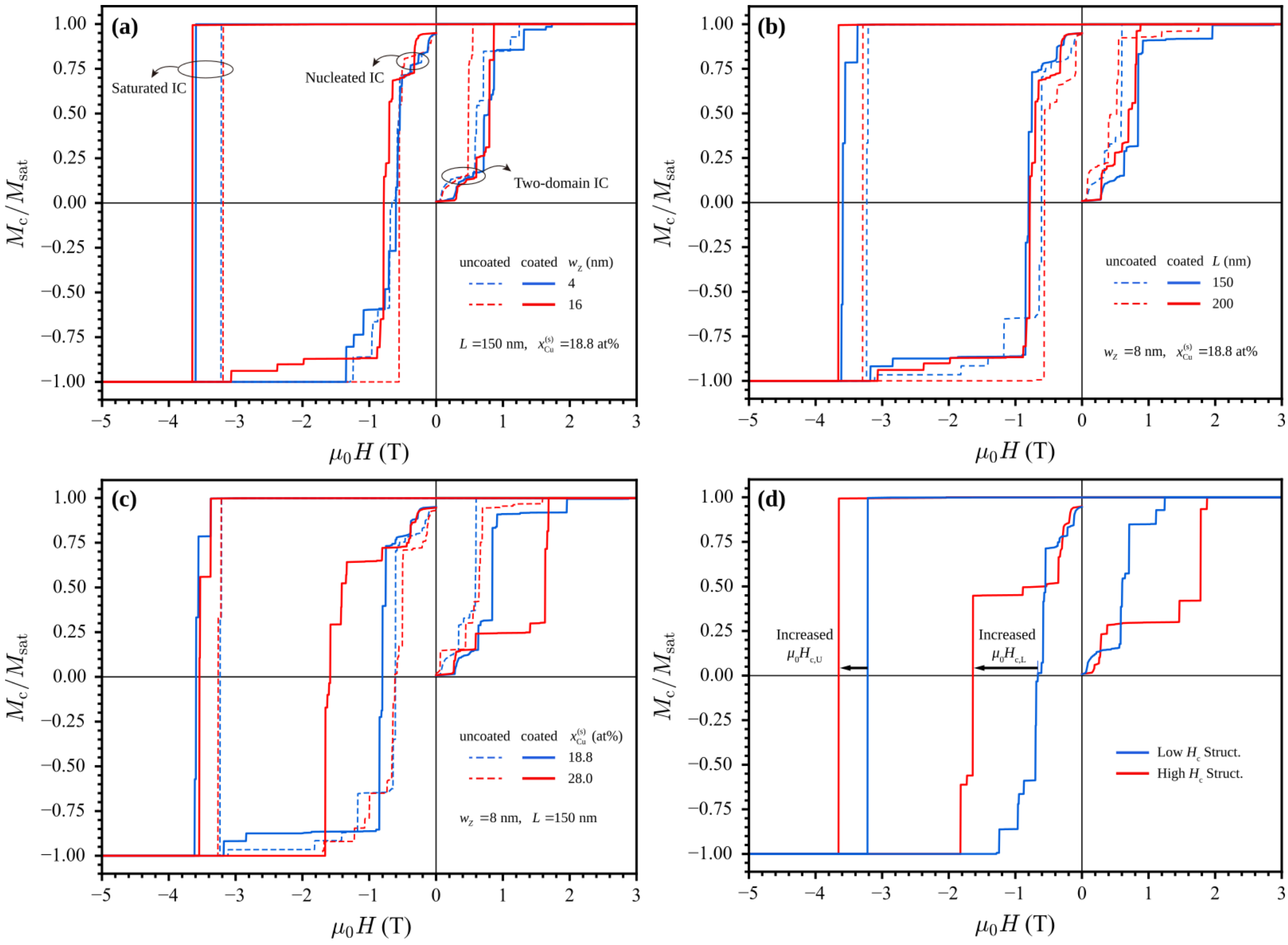

Fig. S11 Simulated initial magnetization and demagnetization curves for three different initial conditions (IC): the parameterized nanostructures each for the 1:5-coated and 1:5-uncoated case **(a)**

varying w_Z **(b)** varying L, **(c)** varying $x_{Cu}^{(s)}$. **(d)** For the proposed high and low coercivity nanostructures also denoting the lower-bound ($\mu_0 H_{c,L}$) and upper-bound coercive field ($\mu_0 H_{c,U}$).

6 Microstructure schematic

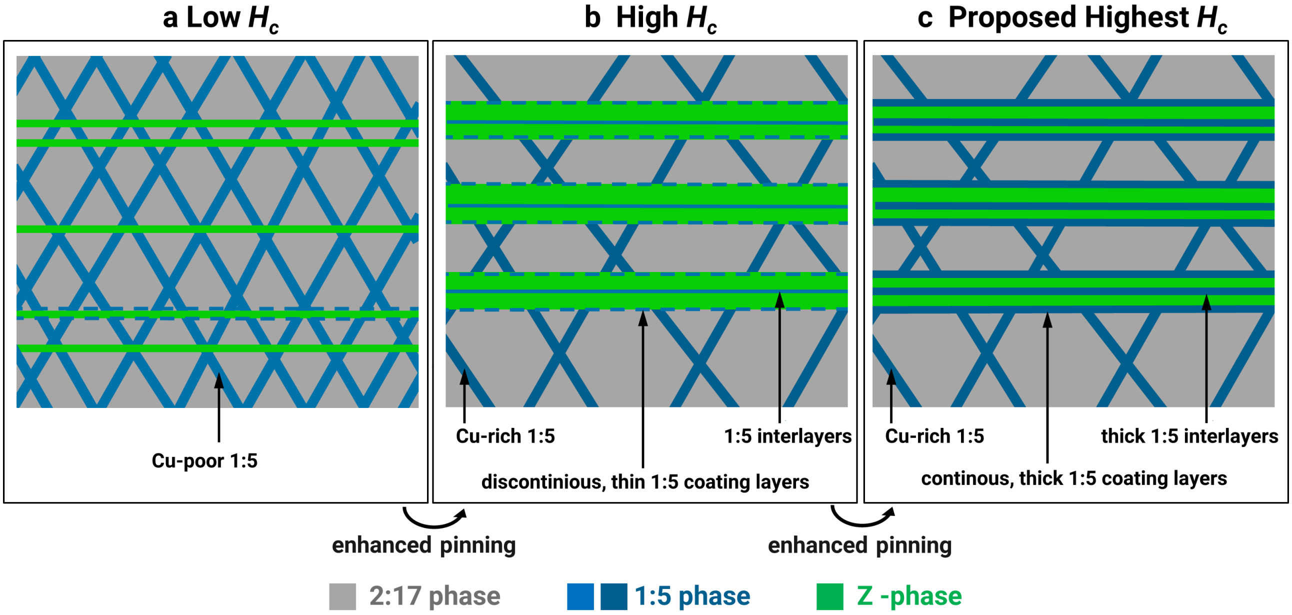

Fig. S12 Microstructure schematics illustrating different regions of magnet A: (**a**) low H_c region, (**b**) High H_c region, and (**c**) Proposed hypothetical microstructure schematics featuring engineered 1:5 coating layers and 1:5 interlayers. These engineered structures are expected to exhibit the highest coercivity compared to the previous configurations, based on the findings of this study.
